# Novel sequence variations in ***LAMA2* and*SGCG* genes modulating cis-acting regulatory elements and RNA secondary structure **

**DOI:** 10.1590/S1415-47572010005000008

**Published:** 2010-03-01

**Authors:** Olfa Siala, Ikhlass Hadj Salem, Abdelaziz Tlili, Imen Ammar, Hanen Belguith, Faiza Fakhfakh

**Affiliations:** Laboratoire de Génétique Moléculaire Humaine, Faculté de Médecine de Sfax, SfaxTunisia

**Keywords:** muscular dystrophies, exon splicing enhancer, RNA fold, SR proteins

## Abstract

In this study, we detected new sequence variations in *LAMA2* and *SGCG* genes in 5 ethnic populations, and analysed their effect on enhancer composition and mRNA structure. PCR amplification and DNA sequencing were performed and followed by bioinformatics analyses using ESEfinder as well as MFOLD software. We found 3 novel sequence variations in the LAMA2 (c.3174+22_23insAT and c.6085 +12delA) and *SGCG* (c. ^*^ 102A/C) genes. These variations were present in 210 tested healthy controls from Tunisian, Moroccan, Algerian, Lebanese and French populations suggesting that they represent novel polymorphisms within *LAMA2* and *SGCG* genes sequences. ESEfinder showed that the c. ^*^ 102A/C substitution created a new exon splicing enhancer in the 3'UTR of *SGCG* genes, whereas the c.6085 +12delA deletion was situated in the base pairing region between *LAMA2* mRNA and the U1snRNA spliceosomal components. The RNA structure analyses showed that both variations modulated RNA secondary structure. Our results are suggestive of correlations between mRNA folding and the recruitment of spliceosomal components mediating splicing, including SR proteins. The contribution of common sequence variations to mRNA structural and functional diversity will contribute to a better study of gene expression.

## Introduction

Whole-genome sequencing of many organisms is producing enormous amounts of data, useful in elucidating the transcriptional regulatory mechanisms of genes. Moreover, comparative sequence analysis of non-coding elements has helped to find new regulatory elements within many genes. New motifs have been discovered from evolutionarily conserved regions (Wasserman *et al.*, 2000), a list of co-regulated genes (Roth *et al.*, 1998), or from a list of functionally related genes (Elkon *et al.*, 2003).

Pre-messenger RNA (mRNA) splicing requires the accurate recognition of splice sites by cellular RNA processing machinery. In addition to sequences that comprise the branch point and the 3' and 5' splice sites, cellular splicing machinery relies on additional information in the form of exonic and intronic splicing enhancer and silencer sequences (Zhang *et al.*, 2009). Splicing enhancers are discrete sequences of 6 or 8 nucleotides that promote both constitutive and regulated splicing, and facilitate exon definition by assisting in the recruitment of splicing factors, especially SR proteins (serine/ arginine-rich proteins) (Chasin, 2007). Ominously, recent studies predict that many human genetic diseases linked to genetic polymorphisms might be caused by the inactivation of splicing enhancers (Wang *et al.*, 2005).

Single nucleotide polymorphisms (SNPs) represent the most frequent type of DNA sequence variations that cause phenotypic variability through multiple mechanisms, this including changes in the encoded protein sequence, the effect on gene regulation, mRNA processing (splicing, mRNA modification and turnover), and translation (Nielsen *et al.*, 2007). The last few years have seen extensive efforts to catalogue human genetic variations and correlate them with phenotypic differences and gene expression. Studies on the impact of genetic variation in mRNA processing have not been sufficiently investigated, especially as it has been documented that a number of neutral genetic variations alter or create essential sequence elements for splicing and mRNA processing (Byrne *et al.*, 2009). These seemingly neutral variations are associated with altered length and/or the steady-state level of cytoplasmic mRNA (Zhang *et al.*, 2007; Hofmann *et al.*, 2008).

We studied the *LAMA2* and *SGCG* genes responsible for the MDC1A and LGMD2C forms of muscular dystrophies, respectively. Limb girdle muscular dystrophies (LGMD(s)) include a heterogeneous group of progressive muscular dystrophy mainly affecting the pelvic and shoulder girdle musculature. 50% of LGMD cases are sarcoglycanopathies related to mutations in *SGCA*, *SGCB*, *SGCD* and *SGCG* genes, thereby leading to the LGMD2D, LGMD2E, LGMD2F and LGMD2C forms respectively (Guglieri *et al.*, 2005; Daniele *et al.*, 2007). Mutations in *LAMA2* gene are known to be involved in the MDC1A form representing that which is most frequent in cases of congenital muscular dystrophy. MDC1A is generally characterized by Total deficiency laminin α2 encoding by the *LAMA2* gene, this leading to a severe phenotype marked by neonatal generalized hypotonia and weakness, with no independent ambulation due to severe contracture (Tome *et al.*, 1994; Helbling-Leclerc *et al.*, 1995). Moreover, over the latter years and from recent studies, there is proof of the presence of mutations in the *LAMA2* gene in some late-onset LGMD forms, where mutations do not result in the complete absence of laminin α2, but in the production of truncated proteins or in increased proteolytic degradation (Naom *et al.*, 1998).

We report here three novel homozygous sequence variations found in the setting of an extensive sequencing of *LAMA2* and *SGCG* genes in two Tunisian patients with autosomic recessive LGMD. These variations were found in 210 tested controls from five different Mediterranean populations (Tunisian, Moroccan, Algerian, Lebanese and French). Two of these variations modulated cis-acting regulatory elements of *LAMA2* and *SGCG* genes and have potential to modify their RNA secondary structure.

## Subjects and Methods 

###  Subjects

New sequence variations were screened in 62 unrelated healthy Tunisian individuals, 50 unrelated controls from Morocco, 45 unrelated healthy controls from Algeria, 35 controls from Lebanon and 20 healthy French individuals. Blood samples were collected after receiving informed consent from all subjects and with the approval of the appropriate Ethic Committees.

###  DNA extraction

Total genomic DNA was isolated from blood leucocytes samples of the tested individuals, according to the previously described protocol of Kawasaki (1990).

###  PCR amplification and DNA sequencing of *LAMA2* and *SGCG* genes

PCR amplification of the 65 exons of the *LAMA2* gene was performed using appropriately chosen primers, so that at least 30 to 50 bp of flanking intronic sequences became readable. For this, a thermal cycler (GeneAmp PCR system 9700, Applied Biosystem) was used, applying the touchdown method as previously described (Guicheney *et al.*, 1998). The 8 *SGCG* gene exons were amplified using primers from LMDP (Leiden Muscular Dystrophy Pages), under optimized PCR conditions consisting of 0.1 μg of genomic DNA, 5 μL of 10 X buffer (50 mM Tris-HCl, pH 9.2, 160 mM (NH4)_2_ SO4, 22.5 mM MgCl_2_, 2% DMSO, and 1% Tween 20), 10 mM dNTP, 20 pmol of each primer and 2U of Taq DNA polymerase. Direct sequencing of PCR products was performed with the ABI Prism Big Dye terminator cycle sequencing Ready Reaction Kit (ABI Prism/ PE Biosystems), and the products were resolved on ABI Prism 3100-Avant. Blast searches were performed using the NCBI database.

###  Computational analyses

Bioinformatic web-based tools were used for predicting the effect of the new detected variations on enhancer composition and RNA secondary structure of the *LAMA2* and *SGCG* genes. Two distinct softwares for analyzing splicing enhancers in detected polymorphisms were employed. In fact, ESEfinder 3.0 (Cartegni *et al.*, 2003), was specifically designed for identifying exonic splicing enhancers, and we used it for the c.*102A/C situated in the 3'UTR which is an exonic untranslated region. Whereas for the other c.3174+22_23insAT and c.6085 +12delA polymorphisms which are situated in intronic regions (intron 22 and 42 of the *LAMA2* gene, respectively); we used the enhancerfinder program (Cartegni *et al.*, 2003). However, the positive and negative results are independent of the software used. Prediction of their effect on the RNA secondary structure was through the MFOLD program (Zuker, 1994).

## Results

###  Novel sequence variations in the *LAMA2* and *SGCG* genes

**Figure 1:**
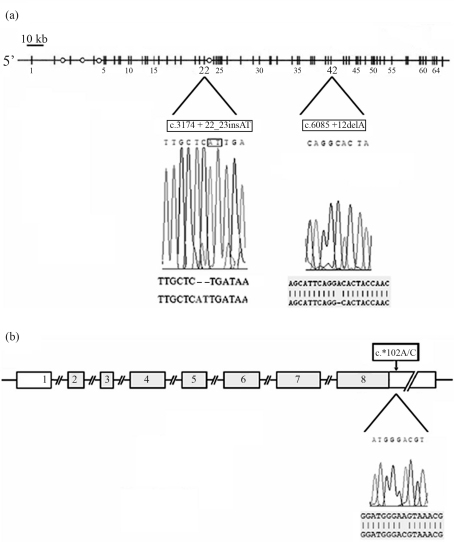
Novel genetic variations in *LAMA2* and *SGCG* genes. Sequencing of the *LAMA2* gene revealed two novel intronic variations: a c.3174+22_23insAT insertion in intron 22 and a c.6085+12delA deletion in intron 42. Sequencing of *SGCG* gene revealed an A > C substitution at position +102 in the 3'UTR region.

Sequencing of the *LAMA2* and *SGCG* genes was performed with the aim to search for sequence variations in two patients with autosomic recessive LGMD. No mutations were found in either gene in the two patients tested. However, novel sequence variations in the *LAMA2* and *SGCG* genes were detected. We revealed the presence of 2 novel homozygous intronic sequence variations in the *LAMA2* gene. The first was an (AT) insertion at position +23 of intron 22 (c.3174+22_23insAT; dbSNP accession number: ss 142460322), whereas the second was a deletion of an adenin at position +12 of intron 42 of the *LAMA2* gene (c.6085 +12delA; dbSNP accession number: ss 142460323) (Figure 1a). In the *SGCG* gene, a new homozygous A to C substitution at position +102 in the 3'UTR region was revealed (c.^*^102A/C; dbSNP accession number: ss 142460324). These sequences were different from those published in the last release of the NCBI GenBank (Figure 1b). Screening of these variations in the *LAMA2* and *SGCG* genes in 60 unrelated healthy Tunisian controls from different regions revealed their presence in the homozygous state in all the tested subjects (Table 1).

This result prompted us to search for these sequence variations on four other ethnic populations. We screened two populations from North Africa, this including 50 healthy Moroccan and 45 Algerian controls, besides individuals from two other Mediterranen populations, namely 35 Libanese and 20 healthy French controls. We found that 100% of the healthy individuals tested were homozygous for the three sequence variations (Table 1).

###  Effect of sequence variations on cis-acting regulatory elements

**Figure 2:**
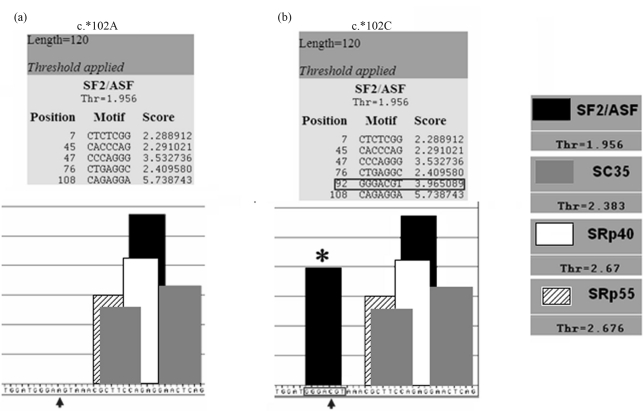
Computational prediction of the effect of c.^*^102A/C change on enhancer composition. (a) The A at position +102 of the 3'UTR of the *SGCG* gene does not include an ESE. (b) Note that the A to C substitution creates a new enhancer in the 3'UTR of the *SGCG* gene recognized by the SF2/ASF SR protein. The arrows indicate the SNP position.

**Figure 3:**
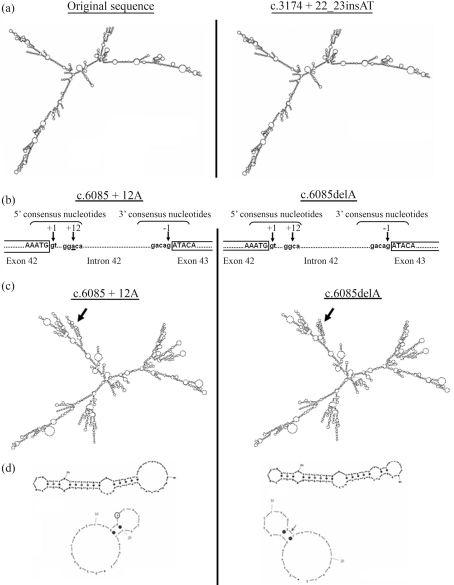
Proposed secondary-structure models of the new sequence variations in *LAMA2* genes. (a) The c.3174+22_23insAT in intron 22 of the *LAMA2* gene was predicted not to affect RNA secondary structure. (b) Position of the c.6085delA in *LAMA2*. The deletion is situated at position +12 in the 5' consensus splice site of intron 42. The c.6085delA is underlined. Position +1 indicates the first nucleotide of the 5' splice site of exon 42. (c) Comparison of RNA secondary structure of [exon 42 + intron 42 + exon 43] between the original sequence and the c.6085+12delA bearing sequence. Several structural changes were predicted. The arrows indicate modifications in folding. (d) Detailed RNA structure analysis of the 5' splicing consensus. The original sequence contained an external closing pair between G^12^C^21^ with a hairpin loop between the G^13^ and C^20^ positions containing an A at position 6085. The c.6085+12delA deletion shifts the 8 bp external hairpin loop and induced a change in its nucleotide composition. Moreover, orientation of the external loop was modified.

The ESEfinder program showed that the c. ^*^102A/C substitution is predicted to create an exon splicing enhancer in the 3'UTR of the *SGCG* gene. The new exon splicing enhancer is a GGGACGT which is predicted to be recognized by the SF2/ASF SR protein with a score of 3.96, thus significantly higher than the threshold value of 1.956 (Figure 2). c.3174+22_23insAT and c.6085+12delA intronic variations in the *LAMA2* gene were predicted not to affect enhancer composition. However, the c.6085+12delA was found to be localised in the donor splicing consensus of intron 42, which mediated a base pairing complementarity with the U1snRNA spliceosomal component (Figure 3b).

###  Correlation between cis-acting elements and RNA secondary structure

In order to study whether pre mRNA folding could be influenced by the presence of these sequence variations in the *LAMA2* and *SGCG* genes, we performed bioinformatics analyses using the MFOLD program. The results strongly suggested that the c.3174+22_23insAT in intron 22 of *LAMA2* had no marked effect on RNA structure (Figure 3a). However, RNA structure analyses of intron 42 and flanking exons (exons 42 and 43) showed that the c.6085+12delA in the donor splicing consensus of intron 42 of *LAMA2* (Figure 3b) was followed by several structural changes (Figure 3c). In fact, detailed RNA structure analyses of the 5' splicing consensus showed that the original sequence contained an external closing pair between G^12^C^21^ with a hairpin loop between those G^13^ and C^20^ positions containing an A at position 6085. The c.6085+12delA deletion shifts the 8 bp external hairpin loop between C^6^ and G^13^ positions, and changes its nucleotide composition, when compared to the original sequence. Moreover, the orientation of the external loop was modified (Figure 3d).

**Figure 4:**
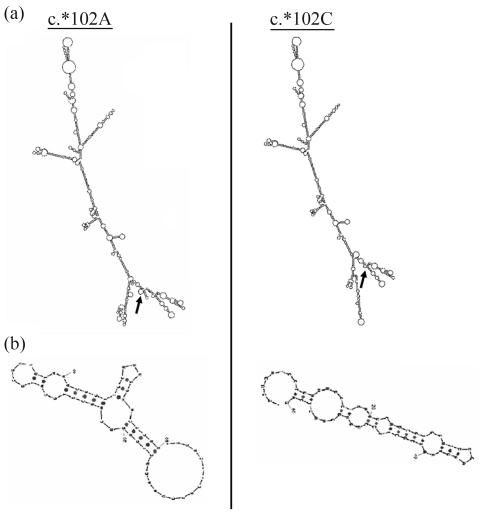
Effect of the c.^*^102A/C in the 3'UTR region of the *SGCG* gene on RNA secondary structure. (a) Analyses of the RNA folding of 500 bp of intron 8 with the whole 3'UTR region of *SGCG*. We marked structural modifications between the c.*102A and the c.*102C alleles. The arrows indicate modifications in RNA folding. (b) Detailed RNA analysis showed that c.^*^102A/C is predicted to create many changes regarding the number, position and orientation of the different external loops.

The creation of an exon splicing enhancer following c. ^*^102A/C substitution in 3'UTRs of *SGCG* is predicted to create many changes in RNA secondary structure, as to the number, position and orientation of the various external loops (Figure 4a, 4b).

## Discussion

The regulatory mechanisms involved in gene expression became an important aspect of genomics revealed by the completed genome sequencing of many organisms.

Most common SNPs have now been assessed in genome-wide studies for statistical associations with many complex traits, these including many important and common diseases (Roeder and Luca, 2009). Although these studies have provided new biological insights, only a limited knowledge of SNPs within regulatory gene elements affecting mRNA post-transcriptional processing has been acquired.

In this study, we discovered three novel sequence variations in the *LAMA2* and *SGCG* genes present in 100% of the 155 healthy tested individuals from North Africa, including Tunisian, Algerian and Moroccan populations and in the 35 and 20 controls from the Lebanon and France, respectively. These results indicate that the c.3174+22_23insAT insertion and c.6085+12delA deletion in *LAMA2*, as well as the c.^*^102A/C substitution in *SGCG* were not only specific to North African, but also apply to other Mediterranean populations. We thus emphasize that these novel sequence variants represent novel polymorphisms in the GenBank sequences of the *LAMA2* and *SGCG* genes.

Intron removal during pre-mRNA splicing in higher eukaryotes requires the accurate identification of the two splice sites at the ends of the exons or in the intron: the exon/intron definition (House and Lynch, 2008). This important task is executed in the nucleus by the spliceosome which is assembled at the correct donor and acceptor splice sites. Moreover, additional information is provided by cis-acting regulatory sequences that serve to enhance or repress splicing, the splicing enhancers and the splicing silencers, respectively (Cartegni *et al.*, 2003).

In our study, the ESEfinder program predicted that c.^*^102A/C created a new enhancer in the 3'UTR of *SGCG*. In the *LAMA2* gene, the c.3174+22_23insAT did not affect the regulatory elements of the gene itself. On the other hand, c.6085+12delA may disrupt base pairing between the donor splicing consensus of intron 42 of *LAMA2* pre mRNA and the 5' end of U1snRNA, this recognition being crucial for U6snRNP recruitment (Lund and Kjems, 2002). The importance of this region was also confirmed in our previous studies which demonstrated that a 7 bp deletion from position +5 to +11 in the donor splicing consensus of intron 17 of the *LAMA2* gene triggered total skipping of exon 17 (Siala *et al.*, 2008). Moreover, the position +12 in the 5' splicing consensus has already been demonstrated to be determinant for the correct mRNA processing of the *ATM* gene through its interaction with the U1snRNA (Lewandowska *et al.*, 2005).

The 3' UTR contains regulatory elements that are essential for appropriate expression of many genes. These regulatory elements are involved in the control of nuclear transport (Ding and Lipshitz, 1993), the polyadenylation status and sub-cellular targeting (St Johnston, 1995), as well as in the rates of translation and degradation of mRNA by the NMD (nonsense mediated mRNA decay) system (Sachs, 1993). These processes are mediated by the interaction of specific sequences in 3' UTRs with specific RNA binding proteins. The creation of an enhancer in the 3'UTR also emphasizes alterations in the splicing pattern and mRNA stability of the *SGCG* transcript. In fact, it could also be responsible of the synthesis of an alternative isoform of *SGCG* mRNA. This idea was already reported in HLA-G transcript (Rousseau *et al.*, 2003) and in the mRNA encoding the β-catalytic subunit of the mitochondrial H^+^-ATP synthase, where translation enhancing activity generates a new isoform, depending upon the cell type analysed (Di Liegro *et al.*, 2000).

These results suggest a possible functional role for both c.6085+12delA and c.^*^102A/C in pre mRNA stability and splicing processing of the *LAMA2* and *SGCG* genes, respectively. The localisation of human coding and non-coding variations in splicing consensus or enhancers, as well as their effect on mRNA splicing has already been reported in several studies (Fairbrother *et al.*, 2004).

The bioinformatics analyses performed to evaluate the effect of these sequence variations on RNA secondary structure showed that the c.3174+22_23insAT is predicted not to affect the RNA structure, whereas the c.6085+12delA insertion in intron 42 of the *LAMA2* gene, as well as the c.^*^102A/C in the 3'UTR of the *SGCG* gene is predicted to generate several alterations in RNA secondary structure compared to the original sequences. This correlation between RNA structure and cis-acting regulatory elements can be explained by pre-mRNA folding within these regions being crucial for their binding to SR proteins (serine/arginine rich proteins), and may be important for the recruitment of spliceosomal components mediating splicing. Indeed, a growing body of evidence has shown that mRNA folding influenced a wide range of transcription events, such as mRNA splicing (Sheng and Tinoco, 1995), processing (Allain *et al.*, 1996), translational control (Pelletier and Sonenberg, 1987) and regulation (Addess *et al.*, 1997).

In conclusion, in this study we reported 3 novel sequence variations in the *LAMA2* and *SGCG* gene sequences, two of which predicted to modulate cis-acting regulatory elements and RNA secondary structure. Sequence variations were retrieved in all the healthy controls tested, thereby indicating their not being involved in disease susceptibility or phenotypic variability. Indeed, the contribution of common sequence variation to mRNA structural and functional diversity could provide an insight into the fundamental mechanisms of gene expression.

## Figures and Tables

**Table 1:** Genotypes of novel SNPs found in the *LAMA2* and *SGCG* genes tested in five ethnic populations.

	*LAMA2* gene (intron 22)			*LAMA2* gene (intron 42)			*SGCG* gene (3'UTR)	
	Original sequence	c.3174+22_23insAT		c.6085 +12A	c.6085 +12delA		c.^*^102A/C	c.^*^102A/C
Tunisian population (n = 60)	0%	100%		0%	100%		0%	100%
Moroccan population (n = 50)	0%	100%		0%	100%		0%	100%
Algerian population (n = 45)	0%	100%		0%	100%		0%	100%
Libanese population (n = 35)	0%	100%		0%	100%		0%	100%
French population (n = 20)	0%	100%		0%	100%		0%	100%

Note that all the tested individuals are homozygous for the novel sequence variations.
